# Regeneration of the skin wound by two different crosslinkers: *In vitro* and *in vivo* studies

**DOI:** 10.22038/ijbms.2024.80137.17361

**Published:** 2025

**Authors:** Naimeh Mahheidari, Morteza Alizadeh, Mohamad Kamalabadi Farahani, Zohreh Arabpour, Nariman Rezaei kolarijani, Ali R. Djalilian, Majid Salehi

**Affiliations:** 1 Student Research Committee, School of Medicine, Shahroud University of Medical Sciences, Shahroud, Iran; 2 Department of Tissue Engineering and Biomaterials, School of Advanced Medical Sciences and Technologies, Hamadan University of Medical Sciences, Hamadan, Iran; 3 Tissue Engineering and stem cells research center, Shahroud University of Medical Sciences, Shahroud, Iran; 4 Department of Ophthalmology and Visual Sciences, University of Illinois, Chicago, IL 60612, USA; 5 Department of Tissue Engineering, School of Medicine, Shahroud University of Medical Sciences, Shahroud, Iran

**Keywords:** Alginate, CaCl_2_, Carboxymethyl cellulose, Glutaraldehyde, Tissue engineering

## Abstract

**Objective(s):**

For designing a suitable hydrogel, two crosslinked Alginate/ Carboxymethyl cellulose (Alg/CMC) hydrogel, using calcium chloride (Ca^2+^) and glutaraldehyde (GA) as crosslinking agents were synthesized and compared.

**Materials and Methods:**

All samples were characterized by Fourier Transform Infrared Spectroscopy (FTIR), Scanning Electron Microscopy (SEM), Blood compatibility (BC), Blood clotting index (BCI), weight loss (WL), water absorption (WA), pH, and Electrochemical Impedance Spectroscopy (EIS). Cell viability and cell migration were investigated using the MTT assay and the wound scratch test, respectively. Besides, the wound healing potential of prepared hydrogels was evaluated on the rat models with full-thickness skin excision. To further investigation, TGF β1, IGF-I, COL1, ACT-A (alfa-SMA), and GAPDH expression levels were also reported by RT-PCR.

**Results:**

Water absorption and weight loss properties were compared between different crosslinker agents, and the most nontoxic crosslinker concentration was determined. We have shown that GA (20 µl/ml) and Ca^2+^ (50 or 75 mM) enhanced the physical stability of Alg-CMC hydrogel, and they are nontoxic and suitable crosslinkers for wound dressing applications. Although *in vivo* assessments indicated that the GA (20 µl/ml) had a cytotoxic effect on tissue repair, Ca^2+^ (75 mM) boosted the wound healing process. Further, RT-PCR results revealed that TGF β1, IGF-I, COL1, ACT-A (alfa-SMA), and GAPDH expression levels were increased in GA (20 µl/ml). Moreover, this trend is the opposite in the Ca^2+^ (75 mM) treatment groups.

**Conclusion:**

This research shows that Ca^2+^ (75 mM) boosts tissue regeneration and wound healing process.

## Introduction

Various biological processes are involved in wound healing and tissue regeneration (1). Despite progress in wound dressing products, skin functional restoration after fulfilled wound healing process is still questionable. This is mostly because of biomaterials limitations and complications. Although they provide a temporary substrate for tissue repair, restoring tissue function is ignored (2). Different biomaterials were developed for tissue repair, including natural and synthetic polymer-based hydrogels (3). 

Although hydrogels possess favorable properties such as water absorption and retention capability, biocompatibility, swelling, biodegradability, and excellent anti-dehydration capacity, they are less controllable for releasing drugs, thus leading to treatment failure (4, 5).

Hydrogel-based wound dressing provides a suitable, moist environment in the wound bed and supports the wound-healing process. Furthermore, there are many wound dressing products based on hydrogels, including ActivHeal® hydrogel, Suprasorb® G hydrogel, AquaDerm™, Gengigel® a gum-care gel based on hyaluronan, Astero®, Cutimed® Gel, DermaFilm®, Gentell CMC Fiber Dressing, Ca-alginate dressing, and NU-Derm™ Hydrogel (6, 7). 

Alginate, extracted from brown algae cell walls, is an anionic heteropolysaccharide composed of a hydrophilic structure with unique properties. More importantly, it is widely used in the form of hydrogel for improving cell survival (8, 9). The mechanical properties of alginate could be improved by the addition of carboxymethyl cellulose (4). Furthermore, ionic and covalent crosslinker-modified hybrid hydrogels composed of alginate combined with carboxymethyl cellulose provide better structure in terms of mechanical properties and cell interactions for tissue regeneration applications (10).

Carboxymethyl cellulose, a major commercial derivative of cellulose and a typical anionic water-soluble polymer, contains many hydrophilic carboxyl groups and a backbone of hydrophobic structure so that it acts as an amphiphilic macromolecule. It is a suitable candidate for wound dressing and medical treatment due to its biodegradability, biocompatibility, and water retention ability characteristics. It is widely used as an emulsifying agent in the cosmetic industry to prevent postoperative adhesions and epidural scarring (11, 12).

In addition, using a single biomaterial among those mentioned above makes fabricating a scaffold that mimics the physiochemical properties of native tissue difficult due to effective crosslinking limitations. Nevertheless, combining multiple biopolymer hydrogels can improve biomaterials’ mechanical, rheological, and biological properties (13).

Crosslinking Alg-CMC is a potentially effective strategy for improving the mechanical strength and physicochemical stability of hybrid hydrogel properties and achieving a new superabsorbent hydrogel (14, 15).

Many crosslinkers, such as physical and chemical crosslinkers, were mainly used to increase the physiochemical structure and stability of hydrogel networks. Physical types include ionic/electrostatic interactions, hydrogen bonding, hydrophobic and crystallization interactions, and ultrasonication techniques. Chemical crosslinkers include photopolymerization, enzyme-based crosslinkers, and “click” chemistry interactions (16, 17). Chemical crosslinkers are preferred to create good mechanical stability; however, the toxicity of the crosslinker agent should also be considered (18). Divalent cationic crosslinkers, including Ca^2+^, Ba^2+^, etc., are considered suitable crosslinkers for alginate-based polymers. They are placed between an α-L-guluronic block of the alginate structure (9). Glutaraldehyde is an excellent chemical crosslinker that is extensively used in medical applications and used to interact with the hydroxyl group of alginate chains by acetyl reaction and a hydrogen bond formation between GA and OH groups of CMC. Although the hydrophilicity of biopolymer networks decreased by crosslinking, their functionality, and mechanical properties were further enhanced (19, 20).

Although these two crosslinkers are commonly used, the most suitable and proper concentrations have yet to be discussed. This work aimed to optimize, characterize, and compare the most suitable and nontoxic concentrations of CaCl2 and glutaraldehyde crosslinkers. Alg-CMC hydrogel was modified with different concentrations of glutaraldehyde and CaCl2 to meet application requirements. The cell toxicity of these two crosslinker agents was compared and evaluated to determine the most suitable concentration of crosslinkers to reach the most effective mechanical properties for further *in vivo* studies. 

## Materials and Methods

***Chemicals***

Sodium alginate (SA), Carboxymethyl Cellulose, Glutaraldehyde (GA) 25 % w/v (AR grade), CaCl2, Dimethyl sulfoxide (DMSO), 3-(4, 5-Dimethylthiazol-2-yl)-2, 5- Diphenyltetrazolium Bromide (MTT), and calcium chloride (CaCl_2_ >97%) were purchased from Sigma-Aldrich (St. Louis MO, USA). Dulbecco’s Modified Eagle Medium: Nutrient Mixture F-12 (DMEM/F-12), Fetal Bovine Serum (FBS), Penicillin-Streptomycin (Pen-Strep), and Trypsin-EDTA were purchased from Gibco (Eggenstein, Germany). 3T3 cells and adult male Wistar rats were obtained from the Pasteur Institute of Iran and Shahroud University of Medical Sciences, respectively.

***Preparation of Alginate-CMC hydrogel***

To prepare Alginate/ Carboxymethyl Cellulose (Alg/CMC) hydrogel, sodium alginate (3 % w/v) and CMC (3 % w/v) were dissolved in distilled water with continuous stirring until a homogeneous viscous solution was obtained separately. The sodium alginate and CMC solutions were mixed in a ratio of 2:1 v/v. Various concentrations of CaCl_2_ (50, 75, 100, 150, 200, and 300) mM and glutaraldehyde (1, 5, 10, 20, 30, and 40) µl/1ml were prepared. Afterward, these mentioned crosslinkers were added to the alginate/CMC solution for crosslinking hydrogels (Table 1). Next, the prepared crosslinked Alg/CMC mixture was stirred at 500 RPM for two hr. Finally, the mixture was kept at -80 °C.

***Characterization of synthesized hydrogels***

*Morphological properties*

The surface morphology of crosslinked Alg-CMC hydrogel samples was observed using Scanning Electron Microscopy (SEM, SERON TECHNOLOGY, AIS2100, South Korea). Prior to completely removing the water, samples were freeze-dried. They were then fixed on carbon stubs and sputter-coated with a thin layer of gold under an accelerating voltage of 20 kV. The pore size of the prepared hydrogel was calculated using Image J software.

*Fourier transform infrared (FTIR) spectroscopy*

FTIR spectra of hydrogels were recorded by FTIR (Spectrum GX, PerkinElmer, USA) from 300 cm^-1^ to 4000 cm^-1^. All freeze-dried hydrogel samples were crushed to 100 mg of KBr and pressed by applying a force of 105 N into a transparent disk. FTIR analyses were conducted after drying the prepared samples at 600 °C.

***Water uptake evaluation***

The water uptake of hydrogel samples was gravimetrically measured at room temperature. At regular time points (1, 3, 6, 12, 24, 48, and 72 hr). Briefly, the predetermined amount of the freeze-dried hydrogels was weighed (W0) and soaked in 10 ml of phosphate-buffered saline solution (PBS, pH = 7.4) at ambient temperature. The samples were extracted and weighted at regular time points to obtain W1. The water uptake rate was calculated using the equation 1:

$\mathrm{Water uptake}=W_{1}-W_{0}/W_{0}\times 100$ Eq. 1

***Weight loss analysis***

Crosslinked Alg-CMC hydrogel samples were freeze-dried for 72 hr. All the samples’ initial weight (W0) was measured and then submerged in 10 ml of PBS. The specimens were removed and dried under vacuum at 70 °C at specific intervals, and the final weight (W1) was observed. The degradation rates were calculated following equation (2).

$\mathrm{weight loss\%}=\frac{W_{0}-W_{1}}{W_{0}}\times 100$ (Eq. 2)

W_0 _is the initial weight of hydrogels, and W_1_ is the dry weight after removal from the water.

***pH analysis test***

The changes in pH of the solution after crosslinked Alg-CMC hydrogel samples were submerged into PBS at different intervals were monitored using a pH meter (S220 SevenCompact, Mettler Toledo). 

***Hemocompatibility evaluation***

*Blood compatibility*

The hemolysis test is an investigational assay extensively used to determine the biocompatibility of treated samples, such as polymeric material. The results are calculated based on releasing hemoglobin content of human RBCs related to material-destroying ability. Initially, 4 ml blood samples were freshly collected from rats into an anticoagulant tube and gently mixed with 5 ml of 0.9% saline. Positive or negative controls, which did not contain hydrogels, were performed by adding 200 µl of diluted human blood to 4.0 ml of distilled water and 0.9% saline solution, respectively. All tests were performed in triplicate by adding 100 µl of each sample to a 96-well plate. Subsequently, add 200 µl of diluted blood into each well. 300 µl of positive and negative control were added to each well separately, and the plate was incubated at 37 °C for one hour. Then, the plate was centrifuged at 1500 rpm for 10 min. Finally, the absorbance of the samples was measured at 545 nm. 

The hemolysis ratio was calculated as described by Dey and Ray (2003) (21) and Mahheidari *et al.* (22) by the following equation, and the average value was reported as the result: 

Hemolysis (%) = Dt-Dnc /Dpc -Dns × 100 % (Eq. 3)

OD of the treated sample (Dt), OD of the negative control (Dnc), OD of the positive control (Dpc)

*Blood clotting index (BCI)*

The blood testing solution was prepared by diluting fresh human blood in a blood tube containing sodium citrate as an anticoagulant to blood ratio of 1:9, v/v. To evaluate the blood coagulation ability of the Alg-CMC samples, each sample was prepared in a 25 ml glass beaker containing 1.5 ml of each crosslinked-hydrogel sample and placed at 37 ^◦^C for 1 hr. Afterward, 100 µl of diluted blood was added to each beaker. After 5 min incubation, 20 µl of 0.2 M CaCl_2_ was added to each sample, and 25 ml of distilled water was added to each sample after 5 min. Then, all samples were transferred to a 96-well plate. Finally, each sample’s optical density (OD) values were measured using a microplate reader at 545 nm. The control group contained 25 ml of distilled and 100 µl of prepared citrated blood, and the average value of triplicated samples was measured in parallel.

According to equation 4, A sample is the absorbance value of each treated sample, and A control is the absorbance value of the control group with no samples. 

BCI = A sample/ A control × 100% (Eq. 4)

*Cell viability measurement*

To evaluate the cytotoxicity effect of CaCl_2_ and GA-crosslinked Alg-CMC hydrogel, an indirect MTT assay was performed according to the manufacturer’s instructions (BIO-IDEA). Mouse embryonic fibroblast cells (3T3 cell lines) were cultured in 96-well culture plates at a density of 1 ×10^4^ cells. After 24 hr, the medium was removed and replaced by 150 μl of media containing (3% W/V) Alg-CMC suspension samples, which were added to a 96-well culture plate at each time point (1, 3 days after cell seeding) and incubated in a humidified atmosphere of 5% CO_2_ at 37 °C for 24 hr. 20 microliters of MTT stock solution (5 mg/ml) was then added to each well and incubated at 37 °C for 3 hr. Afterward, 150 μl of DMSO was added to each well and thoroughly mixed with a pipette. After 15 min of incubation, the absorbance was read at a wavelength of approximately 570 nm using an ELISA microplate reader. Finally, the percentage of cell viability was calculated by equation 5.

Cell viability = OD sample /OD control × 100 (Eq. 5)

*Wound scratch assay*

Cell migration was studied by a wound scratch assay using 3T3 fibroblast cells. The cells were seeded in the 12-well plates at a 5 ×10^4^ cells/well density. After 24 hr of incubation, the culture media was changed. When the cells reached 90% confluency, a scratch was made using a pipette tip of 100 µl. Cells were washed with PBS solution to remove dead cells. The extract of Ca^2+^ and GA crosslinked Alg-CMC samples were placed into each well (n=3 repeated), and then they were allowed to incubate at 37 °C for 24 hr. Images were taken using a time-dependent bright-field Olympus (X53) microscope. Each test was repeated three times for each sample. The wound contraction was calculated using Equation 6.

Wound contraction (%) = (Wd0 - Wdt) /Wd0 * 100 (Eq. 6)

Wd0 is related to the distance between wound boundaries immediately after the wounding procedure, and Wdt refers to the distance between wound boundaries after time “t” of sample treatment.

*Electrochemical impedance spectroscopy*

Impedance measurements of prepared hydrogels were conducted by electrochemical impedance spectroscopy (EIS) by the impedance analyzer (VSP300, Biologic, France) and characterized by applying AC current between a 3*3 cm electrode placed into hydrogel samples and recording the determined voltage between them (applying 10 mV (alternating current)). The values of Re Z (ohms) magnitude, impedance frequency, and Nyquist plot were reported and analyzed by the Zview software. Each measurement of samples was conducted in the range of frequencies from 1 MHz to 7 MHz and applying a programmable interval range voltage of ± 10 mV (alternating current).

*In vivo studies*

Adult male Wistar rats (n=20) with an average weight of about 250 g were obtained from Shahroud University of Medical Sciences. They were housed under standard conditions on a 12/12-hour light/dark cycle at 22.0 ± 0.5 °C, with access to a fed pellet and water tap. *In vivo*, experimental procedures complied with the ethical guideline protocols approved by the Animal Research Ethics Committee of Shahroud Medical University (IR.SHMU.AEC.1402.001). The animals were randomly housed in separate cages and divided into five groups according to the following (n = 4): I: Positive control (PC): No surgery was done; II: Negative control (23): Treated only with sterile gauze; III: Received Alg/CMC treatment; IV: Covered with Alg-CMC-CaCl_2_; V: Received Alg-CMC-GA hydrogel.

To anesthetize animals, 100 mg/kg ketamine and 10 mg/kg xylazine were injected intraperitoneally for each animal. A dorsal full-thickness excision wound at 1.50 × 1.50 cm^2^ was created using a sharp scalpel blade in each rat. Wounds were initially treated with prepared hydrogels (500 µl) in groups (III-V) for each animal, repeated every other day for two weeks.

Ultimately, at the end of 14 days post-surgery, all animals were euthanized using overdosed ketamine (200 mg/kg) administration intraperitoneally, and skin tissue samples at the wound area size were harvested for histopathological investigation.

***Wound closure rate***

Moreover, the wound size of the experimental groups was monitored photographically over time and measured by ImageJ software. The wound closure rate was estimated at different time points (3, 7, 10, and 14 days) by the equation 7: 

Wound closure (%) = (1-) × 100 (Eq. 7)

***Histopathological study***

The excision tissue samples were subjected to 10% buffered formalin to fix and prepare for hematoxylin and eosin staining. Finally, the prepared sections were embedded in paraffin and cut into 5 μm thickness slices. Then hematoxylin and eosin staining was performed, and sections were examined by a light microscope at various magnifications (40x, 100x, and 400x).

*Quantitative real-time polymerase chain reaction (RT-qPCR)*

The total RNA of wound excision tissues was extracted using the RNX-Plus Solution Kit from Iran. Tissue sections were crushed with liquid nitrogen as per the manufacturer’s instructions. Subsequently, cDNA was synthesized with the Easy cDNA Synthesis Kit from Parstous, Iran. The expression of five genes related to wound healing and scar formation was assessed to support histopathological investigations. RT-qPCR analysis was performed with SYBR® Green Real-Time PCR Master Mix from Amplicon, Iran, targeting mRNA levels of TGF β1, IGF-I, COL1, ACT-A (alfa-SMA), and GAPDH (housekeeping gene). The primer details are outlined in Table 2. The relative quantity of mRNA expression was calculated by 2^-ΔΔCt^. All RT-qPCR experiments were investigated using the CFX96 Touch Real-Time PCR Detection System (Biorad). The thermal cycling conditions included an initial denaturation step at 95 °C for 10 min, followed by 40 cycles at 95 °C for 30 sec, 60 °C for 30 sec, and 72 °C for 30 sec. Melting curve analysis of every qPCR was conducted after each cycle. 

***Statistical analysis***

The quantitative data were compared using a one-way ANOVA. Analysis was conducted with GraphPad Prism 5 software. A *P*-value of <0.05 indicated statistical significance. Each experiment was repeated thrice per sample, and data were presented as mean ± SD.

**Table 1:** Various concentrations of CaCl_2_ and GA crosslinked Alg-CMC were investigated in this study

**Samples Alg/CMC (Ratio 2:1)**	**Crosslinker**	**Abbreviation**	**Concentration**	**Volume (ml)**
**1**	CaCl_2_	Alg-CMC- CaCl_2_	50 mM	100 µl
**2**	CaCl_2_	Alg-CMC- CaCl_2_	75 mM	100 µl
**3**	CaCl_2_	Alg-CMC- CaCl_2_	100 mM	100 µl
**4**	CaCl_2_	Alg-CMC- CaCl_2_	150 mM	100 µl
**5**	CaCl_2_	Alg-CMC- CaCl_2_	200 mM	100 µl
**6**	CaCl_2_	Alg-CMC- CaCl_2_	300 mM	100 µl
**7**	GA	Alg-CMC-GA	1 µl	1 µl
**8**	GA	Alg-CMC-GA	5 µl	5 µl
**9**	GA	Alg-CMC-GA	10 µl	10 µl
**10**	GA	Alg-CMC-GA	20 µl	20 µl
**11**	GA	Alg-CMC-GA	30 µl	30 µl
**12**	GA	Alg-CMC-GA	40 µl	40 µl

**Table 2:** Primer sequence used for Real-time PCR to evaluate skin wound healing in the rat model

**Gene**	**Forward sequences (5′–3′)**	**Reverse sequences (5′–3′)**
**rGAPDH**	TCTCTGCTCCTCCCTGTTCTA	ATGAAGGGGTCGTTGATGGC
**rTGF-β1**	AAGAAGTCACCCGCGTGCTA	TGTGTGATGTCTTTGGTTTTGTCA
**rIGF-1**	GCTTTTACTTCAACAAGCCCACA	TCAGCGGAGCACAGTACATC
**rACTA2**	AGCCAGTCGCCATCAGGAAC	GGGAGCATCATCACCAGCAA
**rCOL1A1**	CATGTTCAGCTTTGTGGACCT	GCAGCTGACTTCAGGGATGT

**Figure 1:**
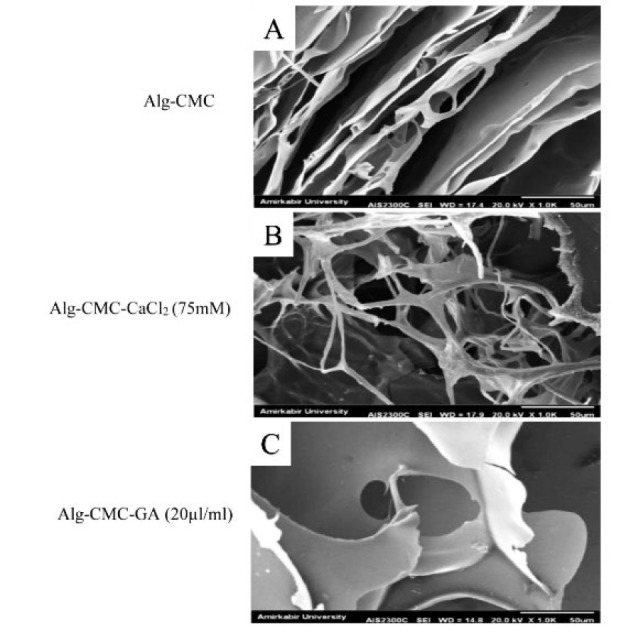
SEM images for morphology characterization of the Alg-CMC (A), Alg-CMC-CaC_l2 _(75 mM) (B), and the Alg-CMC-GA (20 µl/ml) (C) hydrogels

SEM: Scanning electron microscopy; Alg-CMC-CaC_l2_: Alginate-carboxymethyl cellulose - calcium chloride; Alg-CMC-GA: Alginate-carboxymethyl cellulose - glutaraldehyde

**Figure 2:**
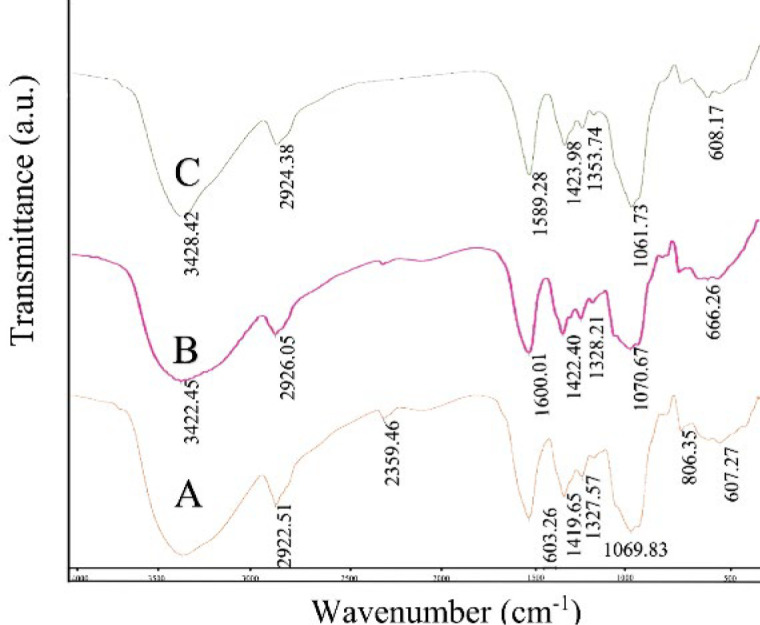
Fourier transform infrared spectroscopy analysis of Alg-CMC (A), Alg-CMC-CaCl_2_ (75 mM) (B) and Alg-CMC-GA (20 µl/ml) (C) hydrogels

Alg-CMC: Alginate-carboxymethyl cellulose; Alg-CMC-CaCl_2_: Alginate-carboxymethyl cellulose - calcium chloride; Alg-CMC-GA: Alginate-carboxymethyl cellulose - glutaraldehyde

**Figure 3:**
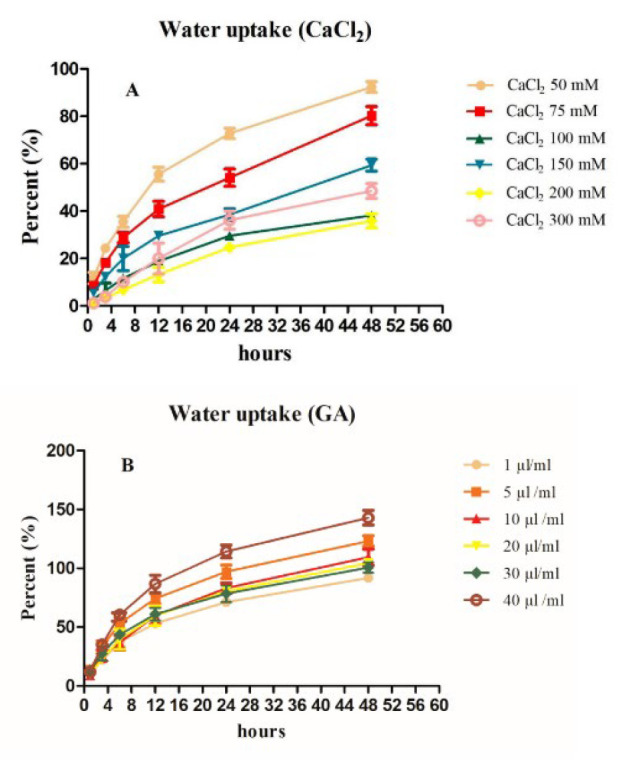
Water uptake results of the prepared hydrogels measured in PBS solution (pH: 7.4) at 37 °C during 48 hr

Water uptake of (A) Alg-CMC-CaCl_2_ concentrations of 50, 75, 100, 150, 200, and 300 mM and (B) Alg-CMC-GA concentrations of 1, 5, 10, 20, 30, and 40 µl/ml at time intervals 1, 3, 6, 12, 24, and 48 hr.

Alg-CMC-CaCl_2_: Alginate-carboxymethyl cellulose - calcium chloride; Alg-CMC-GA: Alginate-carboxymethyl cellulose - glutaraldehyde

**Figure 4:**
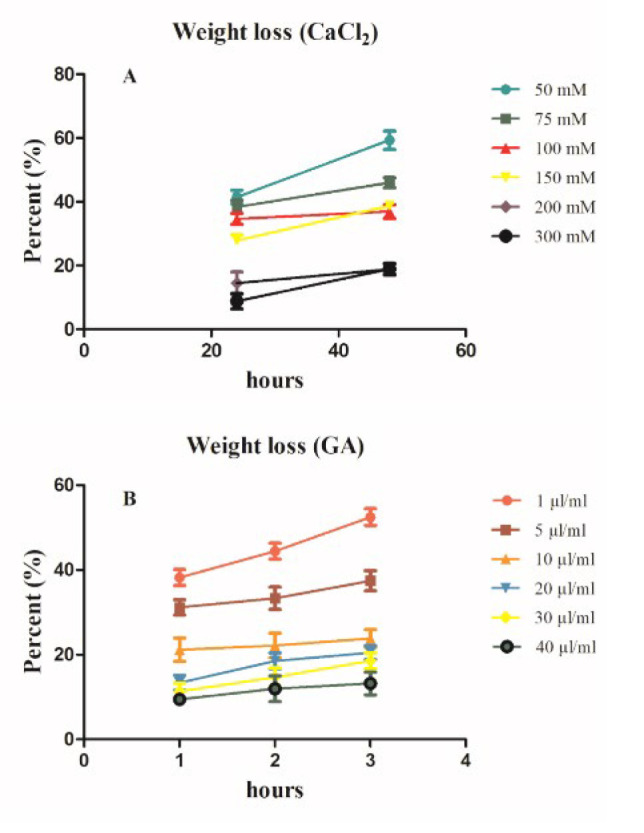
Weight loss results of the prepared Ca^2+^ and GA crosslinked hydrogels measured in PBS solution (pH: 7.4) at 37 °C

Degradation rates of (A) Alg-CMC-CaCl_2_ concentrations of 50, 75, 100, 150, 200, and 300 mM during 48 hr (B) Alg-CMC-GA concentrations of 1, 5, 10, 20, 30, and 40 µl/ml at 24, 48, and 72 hr.

Alg-CMC-CaCl_2_: Alginate-carboxymethyl cellulose - calcium chloride; Alg-CMC-GA: Alginate-carboxymethyl cellulose – glutaraldehyde

**Figure 5:**
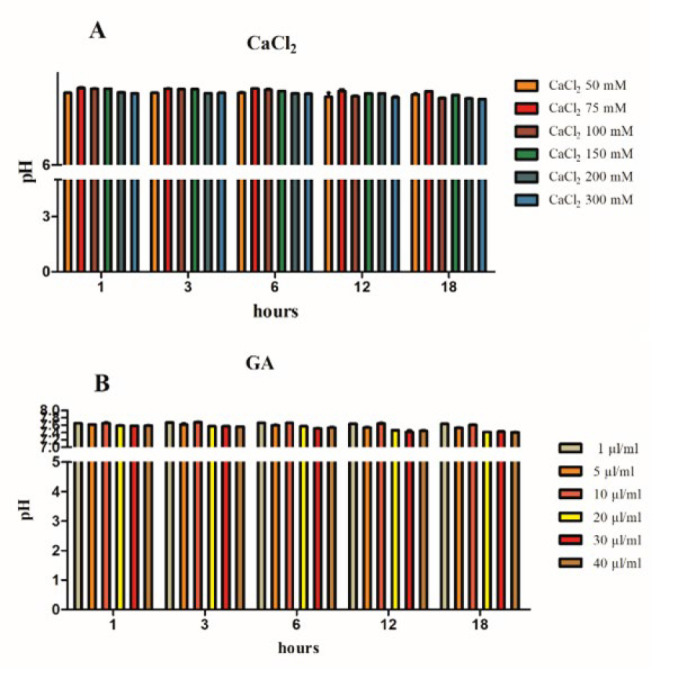
pH changes of hydrogel samples

(A) Alg-CMC-CaCl_2_ concentrations (50, 75, 100, 150, 200, and 300) mM, (B) Alg-CMC-GA concentration series (1, 5, 10, 20, 30, and 40) µl/ml after soaking the hydrogel samples in PBS for different periods

Alg-CMC-CaCl_2_: Alginate-carboxymethyl cellulose - calcium chloride; Alg-CMC-GA: Alginate-carboxymethyl cellulose – glutaraldehyde

**Figure 6:**
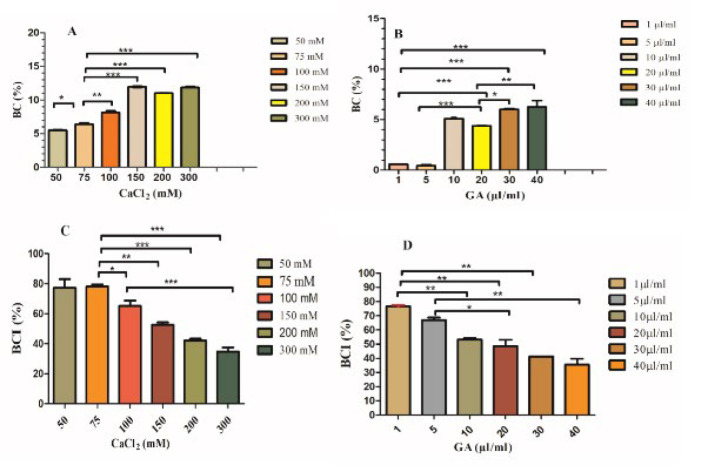
Hemolysis ratio of Alg-CMC-CaCl_2_ samples (50, 75, 100, 150, 200, and 300) mM (A) and Alg-CMC-GA samples (1, 5, 10, 20, 30, and 40) µl/ml (B), blood coagulation index of Alg-CMC-CaCl_2_ samples (50, 75, 100, 150, 200, and 300) mM (C) and Alg-CMC-GA samples (1, 5, 10, 20, 30, and 40) µl/ml (D)

* *P*-value<0.05, **P-value<0.001,***P-value<0.0001

Alg-CMC-CaCl_2:_ Alginate-carboxymethyl cellulose - calcium chloride; Alg-CMC-GA: Alginate-Carboxymethyl Cellulose – Glutaraldehyde

**Figure 7:**
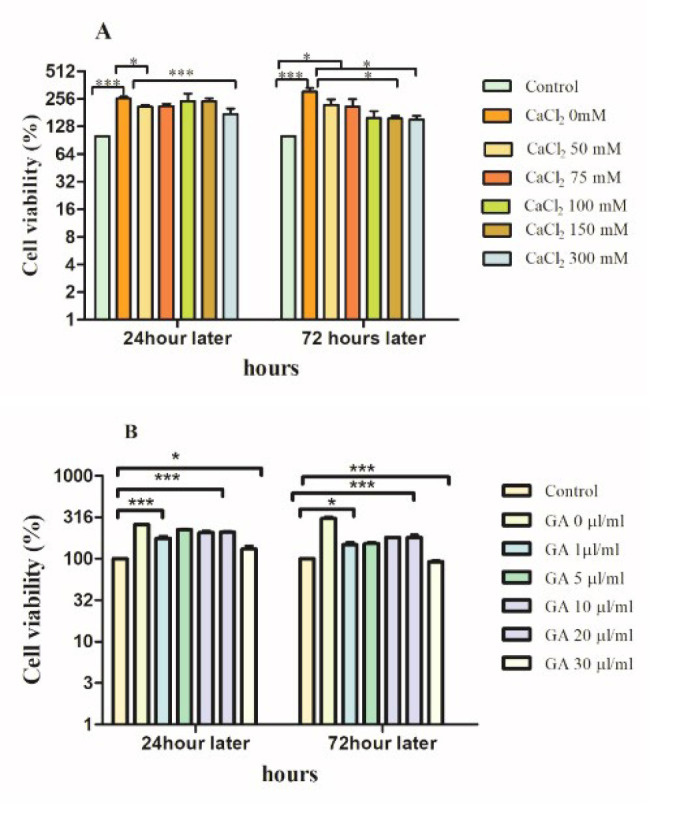
Viability of 3T3 cells of prepared hydrogel samples, evaluated by indirect MTT assay at 24 and 72 hr post-cell seeding

Alg-CMC-CaCl_2_ (50, 75, 100, 150, and 300) mM (A) Alg-CMC-GA (1, 5, 10, 20, 30, and 40) µl/ml (B). Control: Tissue Culture Plastic (TCP). Values represent the mean ± SD, n = 3, *P<0.05, ** *P*<0.01, and *** *P*<0.001

Alg-CMC-CaCl_2_: Alginate-carboxymethyl cellulose - calcium chloride; Alg-CMC-GA: Alginate-carboxymethyl cellulose – glutaraldehyde

**Figure 8:**
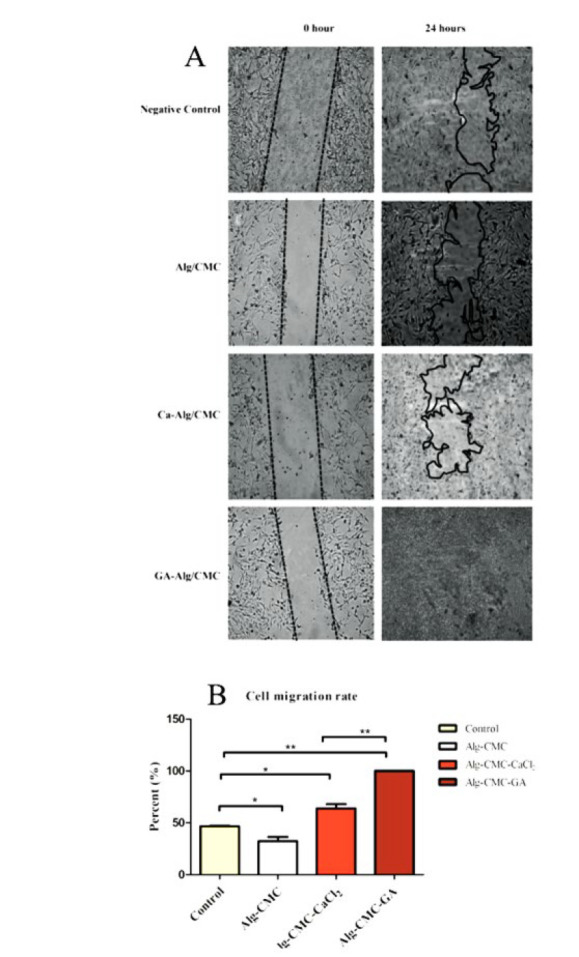
Representative *in vitro* wound scratch assay of 3T3 cell lines treated with Alg/CMC, Alg-CMC-CaCl_2_ (75 mM), and B Alg-CMC-GA (20 µl/ ml) for 0 and 24 hr after treatment

(A) cell migration rate, (B) of Alg/CMC, Alg-CMC-CaCl_2_ (75 mM), and Alg-CMC-GA (20 µl/ml).

Alg/CMC: Alginate-carboxymethyl cellulose, Alg-CMC-CaCl_2_: Alginate-carboxymethyl cellulose - calcium chloride; Alg-CMC-GA: Alginate-carboxymethyl cellulose – glutaraldehyde

**Figure 9:**
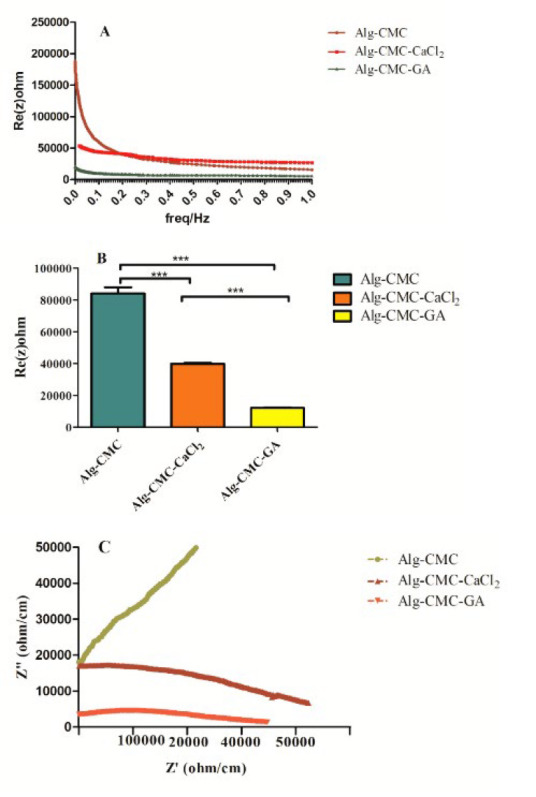
Representative diagram of impedance magnitude (A), resistance comparison (B), and Nyquist plot (C) of pure Alg-CMC, Alg-CMC-CaCl_2 _(75 mM), and Alg-CMC-GA(20 µl/ml)

Alg/CMC: Alginate-carboxymethyl cellulose, Alg-CMC-CaCl_2_: Alginate-carboxymethyl cellulose - calcium chloride; Alg-CMC-GA: Alginate-carboxymethyl cellulose – glutaraldehyde

**Figure 10:**
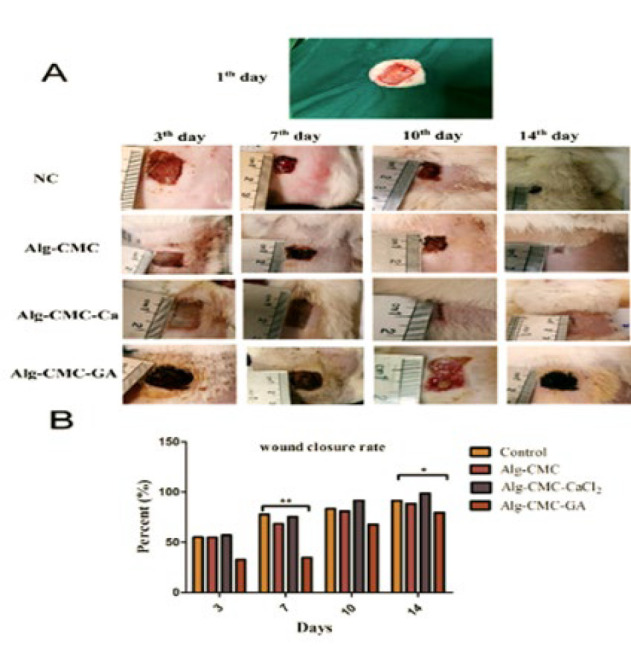
Evaluation of wound healing process macrographical photos of wound healing observation on day 14 treatment by NC, Alg-CMC, Alg-CMC-CaCl_2_, (75 mM), and Alg-CMC-GA (20 µl/ml). (A), Wound closure rates (B) were measured on day 14. (n = 3) *P<0.0, **P<0.01.

NC: Negative control; Alg/CMC: Alginate-carboxymethyl cellulose, Alg-CMC-CaCl_2_: Alginate-carboxymethyl cellulose - calcium chloride; Alg-CMC-GA: Alginate-carboxymethyl cellulose – glutaraldehyde

**Figure 11:**
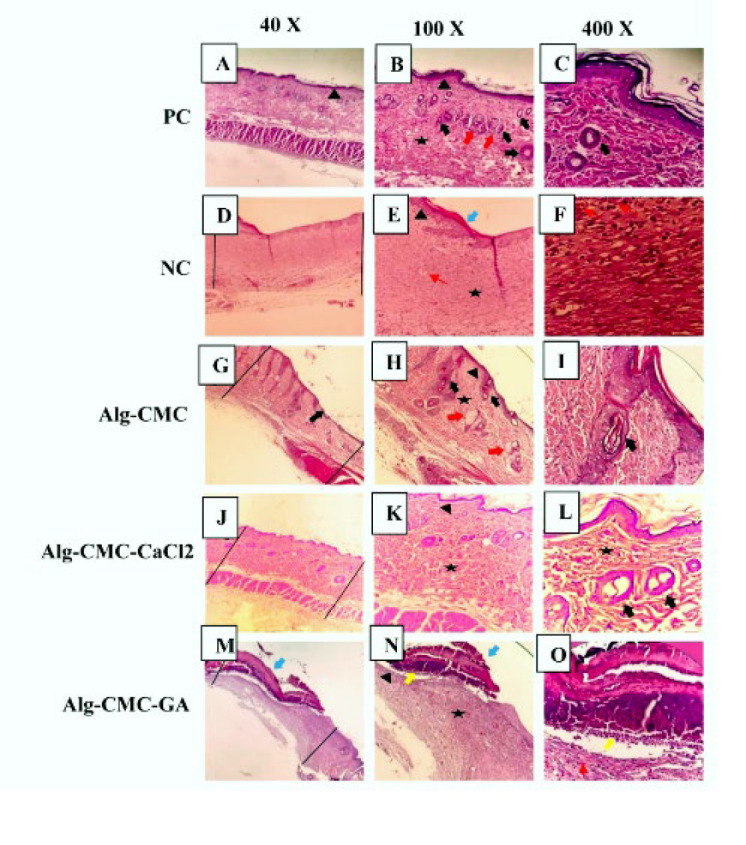
Hematoxylin and eosin (H&E) staining of the skin of rats in different groups of Alg-CMC (G, H, and I), Alg-CMC-CaCl_2_ (J, K, and L), and Alg-CMC-GA (M, N, and O) in comparison with positive control or PC (A, B, and C) and negative control (23) (D, E, and F) on day 14 post-surgery

(Magnification: 40X,100X, and 400X). The new epithelial formation was shown by (black arrowhead ), whereas new blood vessel formation in the dermis was marked by (thin red arrows) and stars refer to collagen formation; thick black arrow and thick red arrow were used for hair follicles and sebaceous glands, respectively. The blue thick arrow and yellow thick arrow demonstrated crusty scabs and inflammatory cells, respectively.

Alg/CMC: Alginate-carboxymethyl cellulose, Alg-CMC-CaCl_2_: Alginate-carboxymethyl cellulose - calcium chloride; Alg-CMC-GA: Alginate-carboxymethyl cellulose – glutaraldehyde

**Figure 12:**
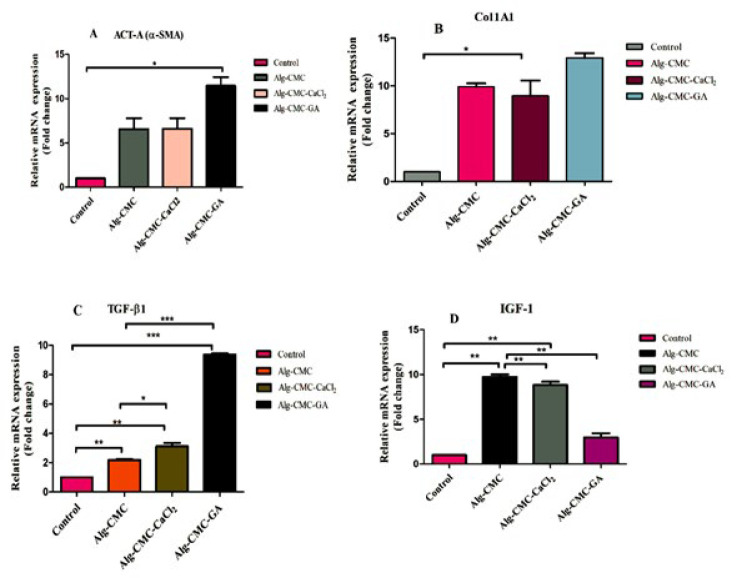
Gene expressions of TGF-βI, CoI1a1, IGF-I, and Act-A (α-SMA) were analyzed by qRT-PCR. Act-A (A), Col1a1(B), TGF-βI(C), and IGF-I (D) in a rat model on 14^th^ day after wound tissue treated with hydrogel samples and negative control

All groups were compared to the control group. Results are demonstrated as mean ± SD. (ns or no significant, *P<0.05, **P<0.01, ***P<0.001).

## Results

***SEM***

To study the surface morphology of the synthesized Alg-CMC, Alg-CMC-CaCl_2_ (75 mM), and Alg-CMC-GA (20 µl/ml), scanning electron microscopy (SEM) was carried out. Figure 1 shows a highly porous and relatively interconnected structure in Alg-CMC-CaCl_2_ (75 mM) and Alg-CMC-GA (20 µl/ml) hydrogels can be observed through the freeze-dry method relative to pure Alg-CMC. Image J software analysis exhibited that the average pore sizes for Alg-CMC-CaCl_2_ (75 mM) and Alg-CMC-GA (20 µl/ml) were 130 µm and 101.75 µm, respectively. Besides, Alg-CMC-GA (20 µl/ml) showed a homogeneous, smoother surface and a smaller pore size than Alg-CMC-CaCl_2_ (75 mM). Although Alg-CMC pore size was observed heterogeneous (Figure 1A) compared to Alg-CMC-CaCl2 (75 mM) (Figure 1B) and Alg-CMC-GA (20 µl/ml) (Figure 1C), the mean of pore size was calculated at 66 µm. Furthermore, the inner layer of Alg-CMC ionically crosslinked with Ca^2+^ was thinner and fibrous than Alg-CMC-GA (20 µl/ml). Also, a high-alignment parallel sheet was obviously obtained in Alg-CMC-GA (20 µl/ml).

***FTIR***

The FTIR spectra of CaCl_2 _(75 mM) and GA (20 µl/ml) crosslinked Alg-CMC hydrogels and non-crosslinked Alg-CMC hydrogels are depicted in Figure 2. The strong bands at 1069.83 and 1070.67 cm^–1^ in non-crosslinked (Figure 2A) and CaCl_2_ (75 mM) crosslinked hydrogel and a strong sharp band around 1061.73 cm^–1 ^in GA (20 µl/ml) crosslinked hydrogels correspond to -C-O-C vibration groups, respectively, and the weak low stretching bands at 1327.53, 1328.21, and 1353.74 cm^–1^ are assigned to -CH_2_ vibration. The similar close absorption peaks from 1580 cm^–1^ to 1603 cm^–1^ in all samples can be attributed to asymmetric vibrations of the C-O-O groups of carboxylic acid. Moreover, the vibration bands at 1419.65, 1422.40, and 1423.98 cm^–1^ refer to the C-H group in all samples.

The absorption peaks in all samples at the 3200-3550 cm^–1^ range are attributed to intermolecular interactions and intramolecular strong hydrogen bonds of O-H groups. Small sharp absorption modes at around 2840-3000 cm^–1^ can be assigned to vibration stretching bonds from C-H groups alkyl groups of alginate attaching to carboxyl functional groups of CMC. The vibration modes at 666.26 cm^-1 ^in Alg-CMC-CaCl_2_ (75 mM) are due to stretching bonds of crosslinking functional groups between Ca^2+^ and carboxyl groups of CMC (Figure 2B). Meanwhile, in the spectrum of the crosslinked Alg-CMC-GA (20 µl/ml) (Figure 2C) and non-crosslinked Alg-CMC the small and wide visible absorption peaks at 608.17 and 607.27 cm^-1 ^belong to the bending mode of O-H groups. Finally, vibration modes at 806.35 cm^-1 ^in non-crosslinked Alg-CMC denoted C-H stretching vibration from mannuronic acid (Figure 2C). 

***Water uptake***

The water absorption rate of hydrogel was measured from its water-absorbing capacity in PBS solution during time intervals after 48 hr. The water absorption of crosslinked hydrogels is shown in Figure 3. The crosslinked hydrogels with GA in various concentrations (Figure 3B) showed an extremely decreased trend by increasing the concentration of CMC to alginate, which contributes to the stability of the covalently crosslinked structure; besides that, swelling behavior decreased. It was suggested that glutaraldehyde could effectively crosslink at the hydroxyl groups of CMC by hydrogen bond interaction (24). The swelling ratio percent of crosslinked hydrogel with various concentrations of Ca^2+^ (Figure 3A) could be slightly decreased when the amount of CaCl_2_ increased regarding the interaction between Ca^2+^ and carboxylate groups in alginate and CMC. Despite a slight increase in all groups during 72 hr, the percent ratios compared to lower concentrations are meaningfully different. Generally, the enhancement in the water absorption ratio can be related to the hydrophilic nature of hydrogel, which is fixed and becomes more stable with higher concentrations of GA and Ca^2+^ crosslinkers. All percent are reported by cumulative frequency. 

***Weight loss***

The degradation rate of hydrogels was calculated from their mass loss in PBS solution for 72 hr. The degradation rates of the prepared Ca^2+^ and GA concentrations based on cumulative frequency percent were evaluated in PBS solution for 72 days. (Figure 4 A&B). The results showed that Ca^2+^ and GA concentrations had a potential effect on the weight loss of the hydrogel. The results indicated that increasing the concentration of crosslinker agents decreased the weight loss percentage of the hydrogel. However, 200 mM and 300 mM CaCl_2 _and 30 µl of GA represented the lowest weight loss of 24.55%, 30.54%, and 17.23%, respectively, compared to other concentrations on the third day. Nevertheless, hydrogel weight loss tended to increase during 72 hr for all series of both Ca^2+^ and GA. Alg-CMC-CaCl_2_ and Alg-CMC-GA percent are reported by cumulative frequency.

***pH test***

A pH test was conducted to assay pH changes of various concentrations of Alg-CMC-CaCl_2_ and Alg-CMC-GA after immersing in PBS solution at pH 7.4 for different time points. (Figure 5A) exhibits the pH changes of PBS after soaking the different concentrations of Ca^2+^ ions (50, 75, 100, 150, 200, and 300) mM and GA in various volumes (1, 5, 10, 20, 30, and 40) µl/ml into PBS for different periods (1, 3, 6, 12, 18 hr). The pH: 7.4 of the PBS solution containing lower concentrations of 50 and 75 mM Ca^2+^ decreased from 7.57 to 7.52 and from 7.68 to 7.60, respectively, during the entire 18 hr. However, the pH of the solution containing higher concentrations of Ca^2+^ (200 and 300 mM) decreased from 7.57 to 7.45 and from 7.55 to 7.43 (Figure 5A). Ph changes of higher concentrations of 20 µl/ml, 30 µl/ml, and 40 µl/ml glutaraldehyde decreased from 7.58 to 7.4 after 18 hr (Figure 5B).

***Blood compatibility and blood coagulation index tests***

Hemolysis is correlated to the relative amounts of hemoglobin released into plasma due to erythrocyte rupture and is directly related to the hemocompatibility of hydrogels. The greater the number of RBCs damaged, the larger the value of the hemolysis rate is. The most desired hemolysis rate (HR) or blood biocompatibility rate for material is below 5% (25). Figure 6A shows hemolysis rates of Alg-CMC-CaCl_2_ concentrations (50, 75, 100, 150, 200, and 300) mM were reported (5.6, 6.6, 8.4, 12.05, 11.06, and 11.7%), respectively. Owing to HR values of 100, 150, 200, and 300 mM of Ca^2+^ being reported at more than 5%, they are not considered proper blood biocompatible materials, whereas 50 mM and 75 mM are more preferred to use due to lower HR rates in comparison with the other concentrations. Among all concentrations above, 50 mM Alg-CMC-CaCl_2_ is closer to 5%. Therefore, it has more blood compatibility potential for *in vivo* studies. (Figure 6B), the blood compatibility rates of GA crosslinked hydrogel samples increased slightly (0.7, 0.7, 3.03, 5.1, 5.1%) by increasing concentrations (1, 5, 10, 20, 30, and 40) µl/ml and then stayed constant at the two highest concentrations. The red blood cell suspensions were light pink or clear, indicating no or slight hemolysis. Hence, all percentages were reported as 5% or below; all concentrations in this study are considered acceptable candidates for further studies.

The blood coagulation index reflects the coagulation effect of the hydrogel. The lower the BCI is, the higher the coagulation activity of the prepared hydrogel (10). (Figure 6C) showed that the blood coagulation index of 50, 75, 100, 150, 200, and 300 mM Alg-CMC-CaCl_2_ tended to decrease sharply, and their values were reported at 83.1, 79.51, 68.75, 54.21, 43.3, and 37.5%, respectively. The results revealed that CaCl_2_ 300 mM could promote the coagulation process more than the others. (Figure 6D) shows hydrogels crosslinked using 1, 5, 10, 20, 30, and 40 µl/ml of GA with BCI values of 75.61, 68.7, 54.21, 53.01, 41.2, and 31.25 %, respectively (n = 3). A trend decline was observed by increasing glutaraldehyde and GA 40 µl/ml concentrations, resulting in the lowest blood coagulation index. Differences in all samples were significant, and blood clotting values in all series were in the acceptable ranges. * *P*-value<0.05, **P-value<0.001, ***P-value<0.0001, ns: non-significant.

***Cell viability (MTT)***

To identify the effect of hydrogel crosslinking via Ca^2+^ on biocompatibility, an MTT assay was conducted to evaluate further the biocompatibility of prepared hydrogel samples on the 3T3 cell line. Hydrogels’ cell viability was measured 24 and 72 hr after cell seeding. As is shown in (Figure 7A) among the prepared Alg-CMC-CaCl_2_ concentrations (50 mM, 100 mM, 150 mM, and 300 mM), all of them did not induce any sign of cytotoxicity in the study of the 3T3 cell line. More importantly, 50 and 75 mM Ca^2+^ showed more cytocompatibility at 24 and 72 hr in comparison with the other concentrations, almost at a high cell viability percent of 150%. Thus, these two concentrations were considered more suitable for the next step of *in vivo* studies. The cytotoxicity of Alg-CMC-GA at various series concentrations (1 µl/ml, 5 µl/ml, 10 µl/ml, 20 µl/ml, 30 µl/ml, and 40 µl/ml) was evaluated in 3T3 cells, and the results indicated that all concentrations were nontoxic; however, 20 µl of GA compared to the other groups showed a higher proliferation rate at both 24 and 72 hr after cell seeding with more than 100% cell viability. (Figure 7B) showed that hydrogel samples are not only biocompatible but could also promote cell proliferation rate in an obvious dose-dependent manner at 24 and 72 hr, which were statistically significant compared to the control group. In addition, the cell viability of all groups was statistically higher than the positive control in both time intervals. Generally, the findings of cell viability studies revealed that the crosslinker agents have no negative effect on the cytocompatibility and cell proliferation of the 3T3 cell line. Nevertheless, Ag-CMC-GA demonstrated a decreasing trend toward 3T3 cell line proliferation rate after 72 hr. The proper concentrations of Alg-CMC-GA and Alg-CMC-CaCl_2_ with the highest proliferation rate after three days were selected for further *in vivo* experiments. *P<0.05, ** *P*<0.01, and *** *P*<0.001.

***Wound scratch***

A wound scratch assay was performed to investigate the cell migration behavior of the 3T3 fibroblast line. An *in vitro* wound closure assay was carried out on 3T3 fibroblasts, and a visual macrograph was taken at the regular time points of 0- and 24 hr post-migration. Appropriate concentrations of Alg-CMC-CaCl_2_ (75 mM) and Alg-CMC-GA (20 µl/ml) were determined by MTT assay. After that, Alg-CMC-GA 20 μl/ml and Alg-CMC-CaCl_2_ 75 mM were used to evaluate *in vitro* cell migration of fibroblasts after treatments. Cell migration of 3T3 into the wound area in Alg-CMC-Ca was highly accelerated 24 hr after treatment relative to the control group, as shown in (Figure 8A). It was also higher than the Alg-CMC wound closure area during 24 hr. (Figure 8B) indicates that cell migration of Alg-CMC-GA (20 µl/ml) into the cell-free outlined is remarkably accelerated compared with Alg/CMC and Alg-CMC-CaCl_2_ (75 mM). It is clearly observed that the Alg-CMC-GA gap area took 24 hr to close completely. In the Alg-CMC-GA hydrogel wound area, edges disappeared completely 24 hr post-treatment. The cell migration rate was estimated as the wound area size was reduced.

***EIS***

According to the literature, EIS was performed to evaluate hydrogel conductivity, and the results were reported at 25 °C. It should be noted that conductivity has an inverse relationship with resistance. Generally, hydrogels with lower resistance exhibit higher conductivity (26, 27). (Figure 9A) demonstrated the resistance (Re (z) ohm vs frequency (28)) diagram. Alg-CMC represented a high impedance at the beginning by applying the lowest frequency, whereas it showed a descending trend with increasing frequency.

Overlapping of various resistance values was observed by increasing frequency in Alg-CMC and Alg-CMC-CaCl_2_ hydrogels. Meanwhile, Alg-CMC-CaCl_2_ and Alg-CMC-GA had similar trends in impedance decreasing over similar frequency ranges compared to pure Alg-CMC. Finally, Alg-CMC-GA indicated the lowest impedance among the two other samples. Therefore, the results of Alg-CMC-GA resistance indicated that the conductivity of Alg-CMC properties has improved. (Figure 9B) depicts a comparison of resistance between Alg-CMC, Alg-CMC-CaCl_2_, and Alg-CMC-GA. Notably, it also confirmed the impedance diagram results (Figure 9C). Additionally, the Nyquist plot in Alg-CMC showed no semicircle at high frequency, but in Alg-CMC-CaCl_2_ and Ag-CMC-GA hydrogel, depressed semicircles were obvious. Moreover, in Alg-CMC crosslinked with Ca^2+^ (75 mM), the semicircles of the Nyquist plot declined (lessened) relative to non-crosslinked Alg-CMC. Rather unexpectedly, not only a lessened semicircle in the Nyquist plot of Alg-CMC crosslinked with glutaraldehyde but also the lowest impedance and resistance were observed in Figure 9 a-c compared to pure Alg-CMC and Alg-CMC-CaCl_2_, respectively.

***In vivo wound healing***

Prepared hydrogel wound healing efficacy was observed in rats with full-thickened wounds 3-, 7-, 10-, and 14 days post-surgery. The percentage of wound closure rate was estimated following exposure to Alg-CMC, Alg-CMC-CaCl_2_, and Alg-CMC-GA. Finally, the results were compared with the negative control group (Figure 10 A and B). The observations showed that wound area significantly reduced two weeks after treatments with Alg-CMC and Alg-CMC-CaCl_2_. The wound edge approximately disappeared in the Alg-CMC-CaCl_2_ treatment on the 14^th^ day. Whereas, the wound area in Alg-CMC-GA exhibited a hypertrophic scar on the 14^th^ day and showed an infection appeared on the 10th day (Figure 10A). Additionally, the wound contraction rate indicated in Alg-CMC-CaCl_2_ and Alg-CMC was significantly accelerated at 97.84% and 88.53 %, respectively, more than that of Alg-CMC-GA. Although the results showed an increased rate (79.45%) after two weeks in Alg-CMC-GA, unexpectedly, the wound area was not only completely healed, but a thickness layer also appeared on the 14^th^ day post-treatment (Figure 10B).

***Histological assessment***

Histological analysis of skin tissue samples was collected on day 14 and stained with hematoxylin and eosin (H&E), and macrographs were taken at three different magnifications: 40X, 100X, and 400X, as shown in Figure 11. Generally, rats treated with Alg-CMC (Figure 11G-I) and Alg-CMC-CaCl_2 _(Figure 11 J-L) exhibited excellent new formation of epithelium thickness and recovery of skin appendages as compared to the negative control (23) (Figure 11A-C). Nevertheless, in Alg-CMC, more immature collagen (star) and fewer hair follicles (thick black arrow) were observed relative to Alg-CMC-CaCl_2 _groups. Alg-CMC-CaCl_2_ results showed more similarities to PC (positive control) (Figure 11D-F). 

Besides, epithelial is almost recovered in the negative control group, with no hair follicle formation or collagen deposition (star). However, a few blood vessels (thin red arrows) can be observed. Indeed, in groups treated with Alg-CMC-GA, an abnormal re-epithelization (black arrowhead) formed in a large area of wound healing site on the 14^th ^day is surrounded by a huge crusty scab layer (blue thick arrow); beneath this layer, a cluster of inflammatory cells (yellow thick arrow) appeared (Figure 11M-O). The fragmentation seen in scab formation may be formed through the tissue preparation method. Finally, healing the epidermis and regenerating the new dermis are obvious. 

***Quantitative real-time PCR***

To further confirm the wound healing effects of Alg-CMC, Alg-CMC-CaCl_2_, and Alg-CMC-GA, the expression levels of α-SMA and Col1A1, TGF-β1, and IGF-I genes were investigated by RT-qPCR (Table 2). GAPDH was used as a housekeeping gene in this study. The results were conducted using Prism 5 software, and comparisons between groups were performed using a one-way analysis of variance (29).

Notably, α-SMA is highly expressed in Alg-CMC-GA and exhibited a 6-fold increase in Alg-CMC-CaCl_2_ and Alg-CMC at a similar level relative to control (Figure 12A). Moreover, in Alg-CMC-GA, Alg-CMC-CaCl_2_, and Alg-CMC groups, a 15, 9, and 10-fold increase in the mRNA expression level of Col1A1 relative to control was observed, respectively (Figure 12B). TGF-β1 expressed a two-fold increase in Alg-CMC-GA and a low level of TGF-β1 in Alg-CMC-CaCl_2_ and Alg-CMC relative to control on day 14 post-wounding (Figure 12C). 

Conversely, IGF-I showed a slight increase in Alg-CMC-GA and a drastic expression in Alg-CMC-CaCl_2_ and Alg-CMC groups relative to the control (Figure 12D). All results correlated with histopathological assessments. 

## Discussion

Abundant biomaterials are used as potential agents in favor of wound contraction rate; however, they require crosslinking agents to improve the stability of the structure, which may exhibit negative complications (30). For example, there are various commercial products based on glutaraldehyde crosslinkers. Besides, it is widely used as a clinically potent crosslinker; whether it is safe when used in direct contact with human tissue is not fully understood (31). Moreover, CaCl_2_ is at the top of the list for hydrogel crosslinking agents and is introduced as a safe and biocompatible material (32). This study conducted a comprehensive investigation to understand GA and CaCl_2_ characteristics better. In the present study, we successfully fabricated and compared various concentrations of GA and CaCl_2_ crosslinked bases on Alg-CMC hydrogel. Several *in vitro* and *in vivo* experiments were conducted to introduce the safest and most effective concentrations of CaCl_2_ and GA agents to enhance tissue regeneration. Indeed, alginate, in combination with CMC hydrogel, becomes more stable and has been introduced as a good candidate for tissue engineering applications, but it is not sufficient for clinical applications. However, crosslinker agents are pivotal in constructing a uniform and stable hydride structure. In addition, biocompatibility and cell toxicity are also crucial in wound dressing applications; thus, the most suitable crosslinker was evaluated and suggested for further experiments.

The morphology, surface, and pore size characteristics of hydrogel samples were changed by chemical and ionically occurring interactions between glutaraldehyde functional groups and Ca^2+^ ions and hydroxyl groups of Alg-CMC hydrogel. A previous study confirmed that a good pore size distribution between 75 μm and 135 μm favors cell attachment, proliferation, and migration (33). Therefore, in the present study, Alg-CMC-CaCl_2_ and Alg-CMC-GA obtained more acceptable pore sizes and improved the alignment layer and interconnected porous structure of hydrogel samples compared to pure Alg-CMC. FTIR spectroscopy was conducted to confirm the structure of Alg-CMC, Alg-CMC-CaCl_2_, and Alg-CMC-GA were blended properly by evaluating the interaction of functional groups in prepared hydrogel samples. According to previous studies, the changes in absorption peaks were described below (34, 35). 

In Alg-CMC-CaCl_2_ at various peaks in the range of 1070.67, 1328, 1600, 1422.45, and 3400 cm^-1^, the sharpness of the peaks slightly decreases but becomes wider than that of pure Alg-CMC. Besides, the absorption peak at about 800 cm^-1 ^was absent, explaining the presence of linkage between Ca^2+^ and functional groups in the Alg-CMC hydrogel. Meanwhile, in the spectra of chemically crosslinked using glutaraldehyde, the sharpness and wideness of the peaks in the spectrum above were surprisingly different. The bands become sharper and narrower, demonstrating the existence of aligned linkage between the functional groups in Alg-CMC and Glutaraldehyde, which possess higher aligned functional groups rather than that of pure Alg-CMC and Alg-CMC-CaCl_2_.

The high water uptake indicated that the structure is suitable for absorbing exudate, which is attributed to the stability and pores of the matrix (36). HUA *et al*. reported that the water-absorbing mechanism can be attributed to the ionization of fixed charges in the hydrogel intermolecular network. The chain length between crosslinker agents is related to dose (37). In our study, the highest concentrations of Alg-CMC-CaCl_2_ showed slower trends in water absorption than lower concentrations of Alg-CMC-CaCl_2_ (75 and 50 mM). Meanwhile, among the Alg-CMC-GA series, the GA (40 µl/ml) obtained the highest water uptake value among them, which may be related to the covalent interaction of glutaraldehyde functional groups with hydrogel samples, which possessed more stability than the ionic interaction of Ca^2+^crosslinker agents. The higher ranges could also be investigated to support the results. Salehi *et al*. proved that crosslinked hydrogels with Ca^2+^ and GA affect the water uptake ratio; furthermore, initial swelling is favorable for increasing pores and facilitating cell attachment and proliferation (38). Previous studies support the results of the present study.

The weight loss of the hydrogel was calculated as a function of degradation obtained from its mass loss in PBS based on our previous study. Moreover, CaCl_2_ induced rapid gelation but poor stability, ascribing rapid deposition of calcium ions outside the hydrogel more than inside. The increasing weight loss rate can be related to the hydrophilic nature of Alg-CMC without crosslinkers; however, GA and Ca^2+^ affect the weight loss ratio. In the present study, decreasing the degradation rate by increasing Ca^2+^ and GA crosslinker concentrations may enhance the physical interactions between the polymer network and link to hydrophilic functional groups, so lower free residue by increasing concentrations of crosslinkers may be available to react with water molecules, so the degradation rate is reduced. On the other hand, aldehyde groups from glutaraldehyde and Ca^2+^ can attach carboxyl residues of Alg-CMC and link intermolecular chains, so the results implied that the degradation degree of crosslinked hydrogels decreased with increasing concentrations. 

A good blood compatibility value was calculated by the hemolysis rate. Excellent blood compatibility is related to a less than 5% hemolytic ratio. The hemolysis effect of glutaraldehyde was evaluated, and increasing HR rates were observed with increasing glutaraldehyde concentrations. More RBC lysis rates are related to higher concentrations of glutaraldehyde. The results implied that higher concentrations of glutaraldehyde have a toxic effect on RBCs. Ali and Tayyab evaluated the hemolysis effect of pre-incubated RBCs from humans, buffalo, sheep, and goats with different concentrations of Ca^2+^. The results declared a linear increase in hemolysis percentages, and the maximum rate is also observed in humans rather than the other mammalian species. The remarkable Ca^2+^ and Mg^2+ ^ATPase activities of the erythrocyte membrane were reported in humans. The other reason is mentioned as a difference in the phospholipid content of human RBCs among the other mammalian species. The highest hemolysis rate is reported at 11% (39), similar to the 200 and 300 mM CaCl_2_ hemolysis rates of 11.06 and 11.7 %, respectively, in this study.

It is declared that pH~7.4 is similar to the biological environment. Therefore, it can be considered an advantage to prevent possible inflammatory responses and benefit cell growth. Alginate hydrogel crosslinked using different concentrations of glutaraldehyde was evaluated at different pHs, and the results proved that intermolecular networks are more stable with higher concentrations of glutaraldehyde and maintain pH at 7.4, so it is suggested for use in controlling drug release. Thus, in our study, the ones that can keep pH at 7.4 or closer, longer than the others, are more beneficial to biomedical approaches. At a pH of 7, sodium alginate is converted to polyanions, which formation is ascribed to carboxyl groups in the hydrogels. GTA and Ca^2+^ crosslinked hydrogels are more stable. Rapid relaxation in the center occurs at pH 7.4 of PBS, attributed to anionic hydrogel’s functional group and repulsion tendency (36). 

The blood clotting index is frequently evaluated based on absorbance; after that, a higher absorbance value of hemoglobin is associated with a slower clotting rate and reflects the anti-thrombogenic activity of a biomaterial. Despite this, no similar study discusses the BCI index of different dosages of glutaraldehyde. In the present study, we demonstrated that the higher concentrations of GA indicated a lower rate of BCI value. Kumar *et al*. prepared a hemostatic alginate hydrogel incorporation with calcium and zinc; according to their results, the hydrogel showed a strong capacity for blood clotting with a significant BCI time reduction (40). Our study showed that the highest concentration of Ca^2+^ ions has a better blood coagulation index, attributed to the lowest BCI rate than the others. Ca^2+^ reduced bleeding time. It is said that calcium plays a pivotal role in the blood clotting cascade and is well-known as clotting factor IV.

The MTT assay is often used to evaluate the cell viability and proliferation effects of biomaterial composition and crosslinking agents. Hence, the cytotoxicity of crosslinked hybrid hydrogels on murine fibroblast (3T3) cells was explored by MTT assays. Cytocompatibility evaluation is crucial in wound dressing applications (41). Finally, the effects of the composition and crosslinking method of hybrid hydrogels on murine fibroblast 3T3 cells were explored by viability assays. However, the alginate hydrogel containing specific concentrations of Ca^2+^ had the lowest cell cytotoxicity compared to the other groups. Different concentrations of Ca^2+^ after seven days of cell seeding inhibited keratinocyte proliferation, whereas the Ca^2+^ toxicity effect on fibroblast proliferation was dose-dependent (42). In this research, the MTT assay results indicated that the high concentration of Ca^2+^ had a nontoxic effect on 3T3 cells; however, lower concentrations showed a higher proliferation rate. According to another study, 3T3 proliferated only when calcium was achieved above 0.1 mM, and the maximum activity of calcium was reported at 0.5 mM. The evidence showed that when 3T3 cells reach a specific proliferation rate, a sufficient concentration of extracellular calcium ions activates a “master” reaction, preventing proliferation and cell division at the G1 phase, implying that Ca^2+^ ions in a dose-dependent manner can affect cell viability rates (43). Glutaraldehyde cell toxicity is related to time and concentration. Cell toxicity was evaluated by mitochondrial enzyme activity measurement of glutaraldehyde 2.5 % in concentrations of 0, 0.1, 0.5, 1.0, 5.0, 10, 20, and 40 µl/ml. The results showed that even the highest glutaraldehyde concentration did not reach maximum mitochondrial enzyme inhibition at 4, 8, and 24 hr compared to formaldehyde (44). Our study found that the highest nontoxic and safe concentrations of Alg-CMC-CaCl_2_ and Alg-CMC-GA after 72 hr were 75 mM and 20 µl/ml, respectively. Thus, these concentrations are preferred for *in vivo* studies.

Cell migration behavior is a critical phase in determining the wound healing process rate. Based on this research, Alg-CMC-GA could effectively contribute to cell movement and cell proliferation after 24 hr. Also, results indicated that Alg-CMC-CaCl_2 _facilitated cell migration faster than the control group after 24 hr. The literature confirmed that alginate-based hydrogels prepared a proper environment for the cell movement of keratinocyte and fibroblast cell lines (45). In the current study, in Alg-CMC-GA, after 24 hr, the 3T3 cell migration rate was significantly higher than Alg-CMC-CaCl_2_ and Alg-CMC alone. Therefore, covering all wound scratch areas implied that Alg-CMC-GA has a high wound contraction rate. Although cell viability percent after 24 hr in Alg-CMC-GA was lower than that of Alg-CMC-CaCl_2_, the cell migration rate of Alg-CMC-GA was faster than that of Alg-CMC-CaCl_2_. The data is consistent with histological assessments.

The conductivity behaviors of Alg-CMC, Alg-CMC-CaCl_2_, and Alg-CMC-GA hydrogels were studied by electrical impedance spectroscopy. Based on a previous study, it is claimed that high conductivity is in accordance with hydrogel’s water content and ion content; therefore, these results were associated with water uptake investigations. In our study, the impedance spectroscopy revealed that Alg-CMC-GA and Alg-CMC-CaCl_2_ exhibited remarkable differences in all over frequencies compared to pure Alg-CMC. Fundamentally, the high conductivity of crosslinker agents can be explained by the re-formation of the new interface double-layer following crosslinker interaction with hydrogel functional groups. Notably, impedance values differed in a wider frequency range for Alg-CMC-GA, Alg-CMC-CaCl_2_, and Alg-CMC-GA. Besides, CaCl_2_ crosslinked Alg-CMC hydrogel achieved a high conductivity and led to a low impedance.

A suitable conductive hydrogel is crucial for integration into the host tissue and has the potential for tissue regeneration. There is no resemblance study of various chemical and ionic crosslinker agents on impedance magnitude changes; therefore, more investigations are essential for understanding the mechanism in detail. 

A previous study (46) declared that strong conductivity notably depends on the polymeric network crosslinking structure. In our study, the Alg-CMC-GA hydrogel showed lower impedance related to higher molecular-chain interactions and, thus, higher conductivity than the other hydrogels. 

According to the literature, the interaction of aldehyde groups with amino and hydroxyl functional groups of hydrogel forms a highly active structure (47). This study assumes that Alg-CMC crosslinked with glutaraldehyde acts as a robust conductive network grade and forms a high-resistance tunnel equivalent to resistance in a parallel circuit. Thus, Alg-CMC-GA demonstrated the lowest resistance among the two other hydrogels. 

All results confirmed by SEM characteristics of hydrogels represent sheet structure formation and constitute a diffusion boundary layer equal to an ion-exchange membrane. Similar to our study, previous studies stated that in the Nyquist diagram, a depressed semicircle represents a low redox reaction (48, 49). Based on a previous study (50), the absence of a semicircle in the Nyquist plot corresponds to the low relaxation time and internal capacity behavior. It has also been claimed (51) that a proper conductive hydrogel resembling skin showed biocompatible and cytocompatible properties in contact with skin and epidermis. In contrast, a dry and low-conductive hydrogel is not suitable for skin. Therefore, our study showed that Alg/CMC hydrogels crosslinked with Ca^2+^ and GA increased the hydrogel stability and endowed the hydrogels with remarkable conductivity properties. According to various studies, it can be inferred that a well-balanced ion exchange at the wound bed inhibits bacterial invasion, promotes cell proliferation, and accelerates wound closure rates. 

*In vivo* wound area closure was monitored two weeks after treatments, and micrographic photos were taken on the 3, 7, 10, and 14^th^ days. According to the literature, alginate-based materials promote hemostasis and enhance skin wound closure due to their high biocompatibility. They prepare a suitable environment with high wound healing potential properties at the wound site (52). Furthermore, in another study, Harriger *et al*. demonstrated that murine wound skin treated with a glutaraldehyde crosslinked graft had poor integration with the tissue host and a delayed wound closure rate due to abnormal formation of the epithelial layer and rather hypopigmented epithelium (53). It is also stated that glutaraldehyde (GA), even at a small millimolar concentration integrated with host tissue, closed the Na^+^ channel and inhibited the transport of sodium in frog skin (54). Previous studies confirmed the wound-healing potential of Ca^2+^ ions as a crucial coagulation factor (29, 55). Previous publications correlated with our results. In the current study, the wound contraction rate in hydrogel crosslinked with GA was significantly lower than in CaCl_2_ crosslinked hydrogel. A lower percentage than the negative control was reported on the 14^th^ day of treatments. Conversely, increasing cell migration rate results and nontoxic cell proliferation effect of 20 µl/ml GA, a difference in results obtained from *in vivo* wound contraction rate, may be attributed to the interaction between blocking current channels in the epithelium membrane at the wound bed. It may lead to a hypertrophic scar. Histopathological assessments and tissue analysis at the molecular level corresponded with the *in vivo* wound closure rate.

As declared in previous studies, early wound closure is often associated with substantial collagen deposition, early tissue granulation, and scar formation. Also, inflammatory cells are essential for regulating wound contraction and scar formation (56, 57). In a typical successive wound healing process, at 14 days post-surgical, the wound area margins are dramatically reduced and approximately covered with healthy epithelium (57). Similar to our study, in treated groups with Alg-CMC and CaCl_2_ crosslinked Alg-CMC, on day 14, a healthy thickness of epithelium and hair follicle formation is obvious due to a perfect regeneration of skin tissue. Whereas, in Alg-CMC-GA, scar formation, abnormal thickness of the new epithelium, and an abnormal inflammatory response may be related to a progressive wound healing process and incomplete wound closure. Besides the existence of crusty scabs, there is evidence of scarring formation and impaired wound healing.

To further investigate the effects of hydrogels on wound closure and the cutaneous wound healing process, the gene expression levels of α-SMA, Col1A1, IGF-I, and TGF-β1 were evaluated by RT-PCR. 

According to the literature, α-smooth muscle actin (α-SMA) is identified as a well-known myofibroblast marker. It is discovered in delayed wound contractions following prolonged inflammation. Higher levels of α-SMA are substantially correlated with thicker scar formation (58). 

On post-surgery day 14, after wound contraction completion and new healthy epithelial recovery, α-SMA and collagen type Ι decreased significantly. α-SMA is correlated with collagen type Ι (59). In our work, Alg-CMC-GA exhibited a high level of α-SMA on day 14 post-operation, indicating a low rate of wound contraction. However, in Alg-CMC-CaCl_2_, there are lower levels of α-SMA. Also, α-SMA is associated with collagen type Ι, indicating early wound closure. The severe expression of Col1A1 represents a progressive wound that heals a thick scab (60). Similar to our study, the expression of Col1A1 and α-SMA in the Alg-CMC-GA group increased.

IGF-I accelerates cutaneous wound healing and drastically increases on day 14 when wound closure is completed (61, 62). In our study, wound closure in Alg-CMC-GA was not fulfilled perfectly, and IGF-I exhibited a slight increase compared to the control. However, in Alg-CMC and Alg-CMC-CaCl_2_, a drastic increase compared to control is related to perfect wound site closure. 

Fibrogenic cytokines, or transforming growth factors (TGF-β1), regulate extracellular matrix (ECM) regulators. Two weeks post-surgery, TGF-β1 levels reduced; however, in early wound closure and hypertrophic scar formation, TGF-β1 expression is significant (63). A decreased level of TGF-β1 represents increasing wound closure rates, consequently restricting type I collagen deposition following myofibroblast differentiation inhibition (64).

In our study in Alg-CMC-GA, the TGF-β1 expression level remained high compared to the control, which may be attributed to impaired wound closure and scar formation. Meanwhile, in Alg-CMC and Alg-CMC-CaCl_2_, the level of expression of TGF-β1 was relatively low due to complete tissue recovery.

## Conclusion

In this work, we compared the two most common commercially available agents to evaluate their wound-healing potential. In this research, CaCl_2_ and GA were used to crosslink Alg-CMC hydrogel to suggest more suitable, nontoxic, and biocompatible concentrations for further experiments in tissue engineering and clinical application. Moreover, in this work, MTT and wound scratch assays exhibited cytocompatibility of 75 mM CaCl_2_ and 20 µl/ml GA crosslinked hydrogel. However, an *in vivo* study on full-thickness wounds in the rat model demonstrated the toxic effect of Alg-CMC-GA, and molecular evaluation also confirmed *in vivo* results. Although the results approved the conductivity capability and high-water absorption ability of Alg-CMC-GA, it is not further suggested for therapeutic fields, according to this study’s strong evidence. For all the reasons mentioned above, we explained that GA crosslinkers exhibit severe complications in tissue repair and therefore suggest considering other safe and more potential crosslinker products for tissue regeneration approaches. Nevertheless, our study approved 75 mM CaCl_2_ as a suitable and proper crosslinker agent for experimental research and can be suggested as a safe agent for clinical purposes.

## Data Availability

The authors confirmed that all data supporting the findings in this study are included in the article.

## Declaration

We have not used any AI tools or technologies to prepare this manuscript.
